# Potential Protective Effect of Hesperidin (Vitamin P) against Glyphosate-Induced Spermatogenesis Damage in Male Rats: Biochemical and Histopathological Findings on Reproductive Parameters

**DOI:** 10.3390/life14091190

**Published:** 2024-09-20

**Authors:** Şükrü Güngör, Murat Kırıkkulak, Barış Denk, Mehmet Fuat Gülhan, Özay Güleş, Duygu Budak, Muhammed Enes İnanç, Fatih Avdatek, Deniz Yeni, Umut Taşdemir

**Affiliations:** 1 Department of Reproduction and Artificial Insemination, Faculty of Veterinary Medicine, Burdur Mehmet Akif Ersoy University, Burdur 15030, Türkiye; sukrugungor@mehmetakif.edu.tr (Ş.G.); enesinanc@mehmetakif.edu.tr (M.E.İ.); 2 Department of Reproduction and Artificial Insemination, Faculty of Veterinary Medicine, AfyonKocatepe University, Afyonkarahisar 03200, Türkiye; mkirikkulak@aku.edu.tr (M.K.); favdatek@aku.edu.tr (F.A.); dyeni@aku.edu.tr (D.Y.); 3 Department of Biochemistry, Faculty of Veterinary Medicine, AfyonKocatepe University, Afyonkarahisar 03200, Türkiye; bdenk@aku.edu.tr; 4 Department of Medicinal and Aromatic Plants, Technical Sciences Vocational School, Aksaray University, Aksaray 68100, Türkiye; mfuatgulhan@aksaray.edu.tr; 5 Department of Histology and Embryology, Faculty of Veterinary Medicine, Aydın Adnan Menderes University, Aydın 09016, Türkiye; 6 Department of Zootechnics and Animal Nutrition, Faculty of Veterinary Medicine, Aksaray University, Aksaray 68100, Türkiye; budakduygu@aksaray.edu.tr; 7 Department of Reproduction and Artificial Insemination, Faculty of Veterinary Medicine, Ankara University, Ankara 06110, Türkiye; utasdemir@ankara.edu.tr

**Keywords:** glyphosate toxicity, hesperidin, testis, histopathology, spermatozoa

## Abstract

The aim of this study was to investigate the effectiveness of hesperidin (HES) on testicular histopathological changes, biochemical changes, and semen characteristics in rats exposed to glyphosate (GLP). The control group was given a normal diet devoid of GLP and HES, the HES group was given 100 mg/kg/day HES with the normal diet, the GLP group was given GLP at the LD_50_/10 dose of normal feed, which was 787.85 mg/kg/day, and the GLP + HES group was given normal feed containing 787.85 mg/kg/day LD_50_/10 dose of GLP in addition to 100 mg/kg/day HES. GLP administration reduced sperm motility, sperm plasma membrane integrity, glutathione levels, and total antioxidant levels in the testicular tissues of rats. Moreover, it caused an increase in right testis and left epididymis weights, abnormal sperm counts, malondialdehyde levels, total oxidant status, and DNA damage. The HES treatment showed curative effects on these parameters. Furthermore, HES was effective in lessening the histopathological damage that was caused by GLP. The results showedthat HES protects spermatological parameters and DNA integrity, improves antioxidant defenses, and lowers the damage and lipid peroxidation caused by GLP in testicular tissue.

## 1. Introduction

In recent years, glyphosate (GLP) and its commercial formulations, like Roundup^®^, have drawn more attention due to the harm they cause to reproductive systems. Even at low concentrations, GLP reduces the effect of the enzyme aromatase that is responsible for converting androgen to estrogen in human placental cells and embryonic cells [1]. Oxidation, apoptosis, and inflammation are all major harmful side effects of GLP. Studies revealed that GLP has a significant impact on cellular antioxidant levels, resulting in glutathione depletion, enzyme disruptions, and elevated lipid peroxidation in keratinocytes [2,3]. A previous study showed that GLP caused abnormal sperm and damaged testicular tissue by increasing DNA damage and oxidative stress [4]. Oxidative stress occurs as a result of the imbalance between free radicals and antioxidants in the organism. Non-enzymatic antioxidants, such as hesperidin, protect cells by directly neutralizing reactive oxygen species (ROS) and free radicals. These compounds, which include vitamins, flavonoids, and other small molecules, interrupt the chain reactions caused by oxidative stress, preventing damage to lipids, proteins, and DNA. Hesperidin (HES), as a flavonoid, scavenges free radicals and enhances cellular antioxidant defenses by stabilizing free radicals, thus reducing oxidative damage to biological membranes and tissues [5,6,7,8].

HES (3′,5,7-trihydroxy-4′-methoxy-flavanone-7-rutinoside; vitamin P) is a flavonoid found in citrus fruits with active pharmacological properties [9]. The substance in question generates a diverse array of pharmacological responses, such as hypolipidemic, anti-inflammatory, anticarcinogenic, and antioxidant effects [10]. Providing HES treatment to rats with experimentally induced varicocele increased seminiferous tubule diameters and seminiferous epithelial heights, while also reducing testicular damage [11]. Furthermore, HES administration reduced the looser and disordered arrangement of spermatogenic cells, as well as vacuolization on the basal lamina of tubules in decabromodiphenyl ether-treated pubertal mice [12].

The review of relevant literature that was conducted for this study revealed no published studies investigating the potential of HES treatment to prevent GLP toxicity in the reproductive system. This study is based on the hypothesis that supplementing HES with potentially toxic substances in the diet will reduce the detrimental effects of these substances on histopathological changes, biochemical changes, and semen characteristics. Therefore, the study aimed to uncover how HES influences histopathological changes, biochemical changes, and semen characteristics in a rat model.

## 2. Materials and Methods

### 2.1. Chemicals

GLP (Knockdown 48 SL) was purchased from the company HEKTAŞ (Kocaeli, Türkiye), and HES was purchased from Sigma-Aldrich (St. Louis, MO, USA).

### 2.2. Experimental Animals and Procedure

Male Wistar Albino rats weighing 170–190 g and aged 2.5–3 months old were included as the animal material. The rats were housed under standard conditions, maintaining a constant temperature of 25 ± 2 °C, with 60–65% humidity, in ventilated rooms, and a 12 h light/dark cycle. The rats (*n* = 7) in the control group (C) received only standard rat chow (Korkuteli Yem, Antalya/Türkiye) and water adlibitum. All procedures were conducted between 10:00 and 11:00 each day. The experimental phase lasted for 56 days, with the animals divided into four groups, each cage containing six rats. The rats in the GLP group (*n* = 7) were administered GLP (Sigma-Aldrich) at the LD_50_/10 dose (787.85 mg/kg) dissolved in saline through gastric gavage. The rats in the HES group (*n* = 7) were administered 100 mg/kg/day of HES (Sigma-Aldrich) liquated in saline through gastric gavage. A dose of 100 mg/kg was chosen for this study according to the findings reported by Rezaeyan et al. [13]. The rats in the GLP + HES group (*n* = 7) were administered 100 mg/kg/day of HES dissolved in saline through gastric gavage after receiving GLP at the LD_50_/10 dose (787.85 mg/kg) [14].

### 2.3. Assessments of Epididymal Spermatozoon Motility

To test motility, 10 µL of sperm and PBS were put on a slide on a 37 °C heating tray, with a cover slide on top. A 10 µL Eppendorf tube (Hamburg, Germany) was utilized to combine 1 mL of the 100 mOsm HOST solution (8.7 g fructose, 4.9 g trisodium citrate, distilled water to 1000 mL, 100 mOsmL^−1^), which had been kept in a water bath at 37 °C, with 1 μL of the semen specimen. After incubation, a small portion of the mixture was removed, yielding smears that were quickly dried. A phase-contrast microscope was used to count 400 spermatozoa. Head, middle piece, tail, and total spermatozoon abnormalities were examined independently and recorded as % [15].

### 2.4. COMET Assay

About 15 × 10^6^ spermatozoa were mixed with low-melting-point agarose at 37 °C and dispersed on slides covered with 1% normal-melting-point agarose. After applying Triton X-100 (1%) lysate solution (Cat. No. 4250-010-01, Trevigen Inc., Gaithersburg, MD, USA) and DL-Dithiothreitol (DTT; 4 mM; Sigma-Aldrich), the temperature was maintained at 4 °C. The samples were incubated at 37 °C overnight with 60 µL of proteinase K (1 mg/mL) (Sigma-Aldrich) to degrade the protein components of the cell membrane, facilitating access to the DNA. They were left in an electrophoresis container with a pre-cooled 300 mM NaOH and 1 mM Na-EDTA (pH 13) solution for 15 min to adapt. At 25 °C for 20 min, a 300 mA and 25 V electric field was used to attract the negatively charged DNA to the anode. After three 5 min rinses in 0.4 M Tris, at pH 7.5, the slides were stained with 5 g/mL ethidium bromide (Sigma-Aldrich). The evaluation of sperm cells was performed using the Comet Score software (TriTek, Version 1.5, Sumerduck, VA, USA). A total of 200 sperm cells were analyzed across six different microscopic areas.

### 2.5. Assessments of Oxidant/Antioxidant Status

Lipid peroxidation was determined using the method of Draper and Hadley, which involves the quantification of malondialdehyde (MDA), a key indicator of lipid peroxidation [16]. Reduced glutathione (GSH), an intracellular antioxidant, was measured spectrophotometrically at 412 nm using Ellman’s technique, and the quantification results were estimated in units of mg/dL [17]. The measurements of total oxidant status (TOS) and total antioxidant status (TAS) were made using a colorimetric test kit (Rel Assay Diagnostics, Gaziantep, Türkiye). These measurements assess the oxidant and antioxidant systems in total, without differentiating between enzymatic and non-enzymatic components. The calculation of the oxidative stress index (OSI) was performed using the formula OSI = (TOS/TAS) × 100, as described in previous studies [18,19].

### 2.6. Estimation of Blood Testosterone

For the determination of blood testosterone levels, rat blood samples collected via exsanguination were transferred into tubes containing EDTA. The samples were then centrifuged (Nüve NF100) at +4 °C, and the resulting plasma was transferred to Eppendorf tubes and stored at −20 °C until analysis. Before testing, the plasma samples were brought to room temperature. Testosterone levels were measured using ELISA kits from BT LAB Bioassay Technology Laboratory (catalog number E0259Ra, Shanghai, China). This kit utilizes a biotin-based, double-antibody sandwich enzyme immunoassay (EIA) method. All parameters were analyzed at a wavelength of 450 nm, and the results were obtained by applying the absorbance values from the device to the calibration curve.

### 2.7. Examination of Histological Parameters

The left testicles of the rats were soaked in Bouin’s solution for 24 h and then embedded in paraffin. Sizes of 300 μm serial sections at a thickness of 6 mm were produced. Staining (Periodic Acid Schiff Reagent) was performed for histomorphometric analyses. Random selection was made for circular stages of tubules VII–VIII [20]. There were one hundred tubules in each segment, and the proportion of stage XIV tubules per hundred was found [21]. The stained sections were graded under a light microscope as follows: 0 = negative, 1 = low, 2 = medium, and 3 = high.

### 2.8. Statistical Analysis

The statistical analysis was performed using IBM Corp.’s SPSS (v.22, New York, NY, USA). The Shapiro–Wilk W test was performed to test the normality of the distribution of the data. Intergroup differences were analyzed using one-way analysis of variance (ANOVA) and Duncan’s post hoc test. Furthermore, the Kruskal–Wallis analysis of variance and the Mann–Whitney U test with Bonferroni correction were used to assess the histological changes. *p*-values below 0.05 were regarded as statistically significant.

## 3. Results

**Figure 1:**
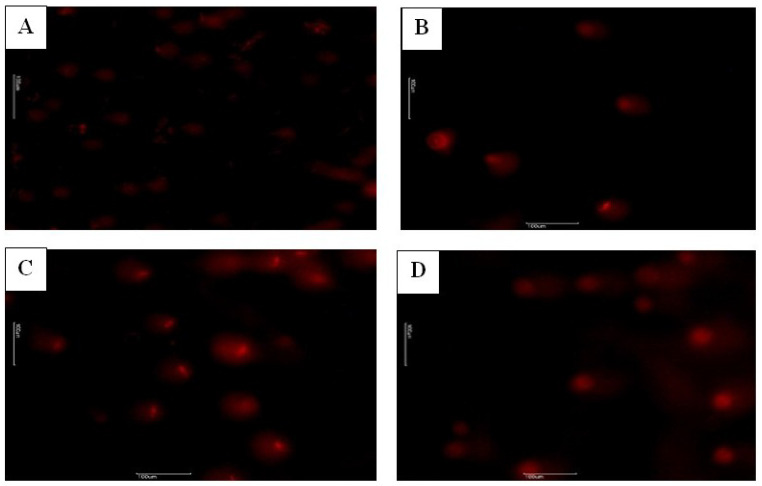
DNA damage in C (**A**), HES (**B**), GLP + HES (**C**), and GLP (**D**) identified using the COMET assay method. Scale bars: 100 μm. C: control, GLP: glyphosate, HES: Vitamin P.

**Figure 2:**
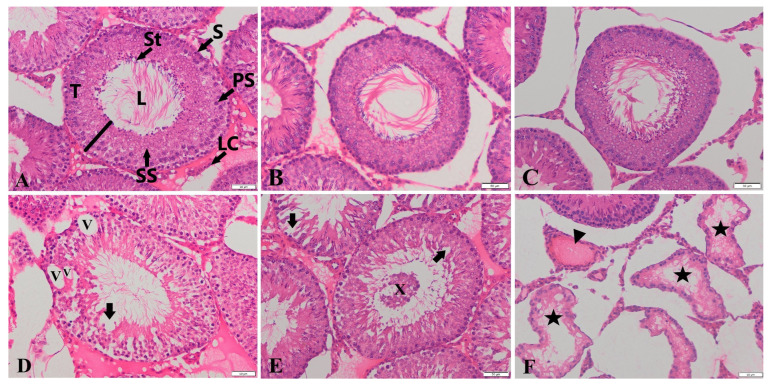
Seminiferous tubules, testicular cross-sections in C (**A**), HES (**B**), GLP + HES (**C**), GLP (**D**–**F**) groups. T: Seminiferous tubule, Black line: seminiferous tubule epithelium, L: lumen, S: spermatogonia, PS: primary spermatocyte, SS: secondary spermatocyte, St: spermatid, LC: Leydig cell, Arrows: germinal epithelium degeneration, V: vacuolization, X: immature germinal cells in the tubular lumen, stars: atrophic seminiferous tubules, triangle: congestion in interstitial blood vessels. Hematoxylin and eosin staining. Scale bars: 50 μm.

**Figure 3:**
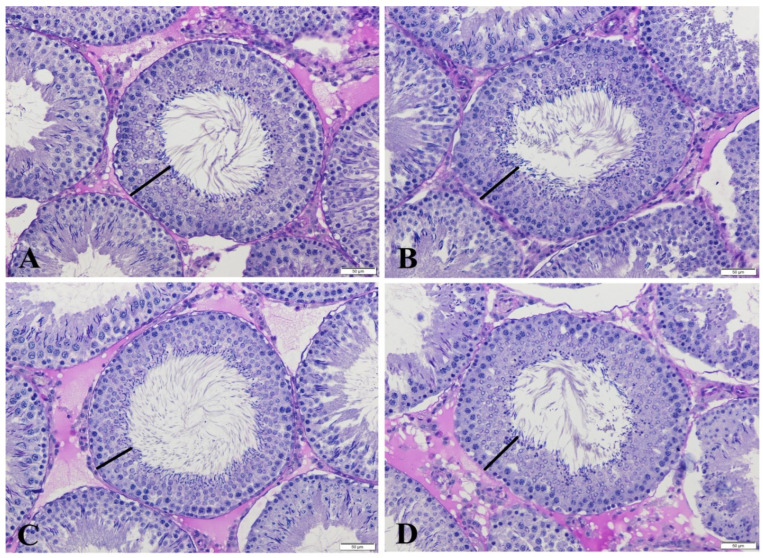
SEHs (lines) in C (**A**), HES (**B**), GLP (**C**), and GLP + HES (**D**) at stages VII–VIII. Black lines: seminiferous tubule epithelium. Periodic Acid Schiff Reagent (PAS) staining method. Scale bars: 50 μm.

As shown Table 1, the rats in the GLP group had lower motility (47.57%) than those in C group (76.42%; *p* < 0.05). This corresponded to a decrease in levels by around 28.85%. Motility was significantly raised to C levels in the GLP + HES group (*p* < 0.05; Table 1). There were no significant differences in testosterone levels between any treatment group and C (*p* > 0.05). Only the HES group exhibited a statistically significant decrease in the number of total abnormal sperm, whereas the GLP group showed a rise compared to the C group (*p* < 0.05). Besides, total abnormal sperm counts decreased to C levels (*p* < 0.05; Table 1) in the GLP + HES group. There were no significant differences in the degrees of plasma membrane integrity (H+/E−) among the groups (Table 2). As shown in Table 3, left testis and right epididymis weights were significantly higher in the GLP and GLP + HES groups than in the C group (*p* < 0.05). In the comparisons of right testis and left epididymis values to C, there was no significant change in any treatment group (*p* > 0.05). MDA, a byproduct formed during the degradation of polyunsaturated lipids, serves as a direct indicator of the extent of lipid peroxidation. Testicular MDA levels rose considerably in the GLP and HES groups compared to C (*p* < 0.05). This metric was lower in the GLP + HES group than it was in the GLP group. Conversely, GSH levels were significantly lower (*p* < 0.05) in every treatment group in comparison to C (Table 4). Table 4 demonstrates that all treatment groups had significantly decreased TAS levels in rat testicular tissues. The TOS levels of the GLP group were significantly higher than those of C and the other treatment groups (*p* < 0.05). In the GLP + HES group, the HES treatment improved DNA damage (*p* < 0.05; Table 5; Figure 1A,C,D). The GLP group showed a significant degree of DNA damage (*p* < 0.05; Figure 1A,D). The HES, C, and GLP + HES groups all had comparable testicular appearances. Examining the testicular tissues in the groups under a microscope allowed the observation of the basement membrane of the seminiferous tubular epithelium, which was composed of germ cells at various developmental stages and had Sertoli cells on it and a normal structure. Additionally, Leydig cells were found in the connective tissues of the inter-tubular space (Figure 2). The rats treated with GLP exhibited more immature germinal cells in the tubular lumen and greater degeneration and vacuolization in the germinal epithelium when their testes were cut open (*p* < 0.001). Furthermore, in the GLP group, there was a significantly greater degree of atrophy in the seminiferous tubules and congestion in the interstitial blood vessels (*p* < 0.001) in comparison to the other groups (Figure 2, Table 6). Compared to the other groups, the administration of GLP decreased the stage XIV tubule rate (%) and STDs at stages VII–VIII. Nevertheless, these reductions were consistent among the groups (*p* > 0.05, Table 7). In comparison to the other groups, the seminiferous tubule epithelial height values in the GLP group considerably declined at stages VII–VIII (*p* < 0.001). Among the C, HES, and GLP + HES groups, there was no significant change in seminiferous tubule epithelial heights at stages VII–VIII (Figure 3, Table 7).

## 4. Discussion

Herbicide exposure affects sperm quantity, motility, and morphology negatively and increases the number of sperm with defects [15]. The results obtained in this study showed that GLP exposure reduced sperm motility whereas it significantly increased abnormal sperm cell counts compared to C. GLP can affect spermatogenesis in animals via tissue-induced free radical production, as sperm counts and motility decrease. These side effects are linked to steroid metabolism changes and antioxidant enzyme inactivity [22]. GLP can alter sperm cell structure as well as testicular DNA integrity. ROS generation impairs mitochondrial energy production, which may explain the reduction in sperm motility [23]. This positive effect of HES presented in this study was interpreted as the prevention of the detrimental effects of GLP on testicular functions in rats due to the antioxidant capabilities of HES. The GLP dosage that was used in this study did not change testosterone levels significantly compared to those in C. The results of some studies similar to those obtained here showed that GLP exposure did not affect testosterone levels [2,24], while others studies reported lowered testosterone levels at concentrations higher than those in this study [25]. Romano et al. [26] found that GLP could reduce Leydig cell population, which could impair testosterone synthesis. GLP competes for steroid receptor binding sites, which may explain its consistent influence on testosterone levels [27]. Thus, the adverse effects of GLP treatment may be due to its unique molecular toxicity pathways against reproductive functions, resulting in identical physiological consequences such as sperm count and motility suppression. HES, a flavonoid known for its potent antioxidant properties, as indicated before [9], likely mitigates GLP-induced damage through several pathways. Specifically, HES may enhance cellular antioxidant defenses by increasing the activity of endogenous antioxidants, thereby neutralizing excess ROS and reducing oxidative stress. Our findings suggested that HES primarily counteracts oxidative damage by stabilizing cellular antioxidants and preventing lipid peroxidation. This protective role of HES is supported by its ability to decrease oxidative stress markers and improve testicular function. Electrophilic radicals produced by xenobiotic metabolism like GLP can increase intracellular ROS production and create free radicals such as singlet oxygen, hydrogen peroxide, and hydroxyl radicals. Enzymatic and non-enzymatic endogenous antioxidant defense systems in cells convert ROS into non-toxic compounds to prevent their surpass. An imbalance in these regulators causes oxidative stress. Lipid peroxidation and oxidative stress induce tissue damage, which raises MDA and TOS levels. On the other hand, ROS attacks on testicular fatty acids increase these indicators. These testes are sensitive to oxidative stress owing to their high composition of polyunsaturated membrane lipids [28]. El-Shenawy [29] reported that TAS, MDA, and TOS levels rose in rat testicles after GLP application, indicating oxidative stress, consistent with the results presented in this study. GLP disrupts the electron transport pathway, releasing free radicals and causing oxidative damage to the testicles. Increased testicular ROS generation alters tissue physiology and can result in DNA damage [30]. GLP can produce oxidative stress by generating ROS, which disrupt antioxidant defense systems and cause cellular damage in tissues like the testes. The GSH redox cycle transforms GSH and lipid peroxides into inert molecules in biological systems [31]. In this study, it was found that GLP dramatically reduced GSH in rat testes. ROS increase oxidative stress, which oxidizes lipids and proteins, lowering GSH levels. Increased lipid peroxide generation inhibits the components of the fat-soluble antioxidant system, including GSH. GSH depletion can impair enzyme functions by rendering GPx and other enzymatic antioxidants more susceptible to oxidative degradation. It was determined that ROS production increased in mice exposed to GLP, causing the severe oxidation of testicular proteins [32]. Thus, the oxidative alteration of proteins may have caused the drop-in enzyme activity in this study. HES dramatically reduced the effects of GLP on oxidant-induced lipid peroxidation and protein breakdown. In this study, a decrease in TAS after GLP administration indicated an overwhelmed antioxidant system in the testes. Male infertility was linked to low antioxidant capacity in male reproductive organs [33]. In this study, the administration of GLP followed by the administration of HES reduced testicular TOS levels. To the best of our knowledge, there are no studies showing that HES protects the testes against GLP-induced oxidative stress, but it has been shown that flavanone has protective effects against reproductive system toxicity. According to Inanc et al. [34], abnormal sperm morphology and excessive ROS production may indicate DNA damage. Oxidative stress activates caspase-8 to trigger apoptosis, which affects spermatogenesis [35]. The obtained results showed that GLP increased testicular DNA damage in rats. Sertoli cells may partly cause GLP-induced testicular changes and germ cell damage, which may substantially affect spermatogenesis. One theory is that GLP upregulates caspases, which induce death in afflicted cells [36]. Enzymatic antioxidants, the initial line of defense against ROS toxicity, work synergistically with exogenous antioxidants with secondary defense mechanisms. Alanbaki et al. [37] found that HES reduced etoposide-induced spermatological dysfunction in rats. The results of another study suggested that this flavonoid compound may reduce testicular function and hormonal axis issues [38]. Flavonoids lower tissue lipid peroxidation, increase antioxidant concentrations, and retain nucleic acids, DNA, RNA, and lipid cellular components in membranes, improving testicular and pituitary functions [6]. In male rats with doxorubicin-induced testicular toxicity, Trivedi et al. [7] showed that HES reduced oxidative stress, DNA damage, and cellular toxicity. The sperm counts and head morphologies were also protected against doxorubicin-induced germ cell damage. Some researchers have used HES to protect the reproductive system from toxic compounds. Kaya et al. [8] determined that HES significantly decreased cisplatin-induced oxidative stress, reducing reproductive system damage. Vijaya Bharathi et al. [22]. found that G-HES, a changed form of HES, protected the reproductive system and nuclear DNA from vanadium toxicity. HES was found to change the ratio of oxidants to antioxidants in rats, reducing benzo(α)pyrene-induced testicular injury [39]. Mammalian apoptosis requires caspase-3. Caspase-3 modulates apoptosis and inflammatory signaling. This enzyme also coordinates DNA fragmentation, cytoskeletal protein breakdown, and cell death. In this study, GLP exposure disrupted the antioxidant defense system, increased testicular lipid peroxidation, and raised protein oxidation. These results were connected to testicular morphology and spermatogenesis changes. A GLP-induced deterioration in rat testicles comprised increased tubular and lumen width and decreased epithelial height according to Romano et al. [26]. Ikpeme et al. [40] found that GLP harms the reproductive physiology parameters of rats, including gonad cellular integrity. In another study, GLP lowered lumen sperm concentration, and resveratrol improved testicular Sertoli cell degeneration [15]. The histology of the rat testes exposed to GLP in this study showed increased embryonic germinal cells in the tubular lumen and a damaged germinal epithelium with vacuoles. Interstitial blood vessel occlusion and seminiferous tubule atrophy also increased considerably in the GLP group in comparison to the other groups. This histological finding demonstrated that GLP exposure increased oxidative stress and reduced antioxidant status. Damage to Leydig cells lowers testosterone synthesis and Sertoli cell testosterone protection [41]. In turn, spermatogenesis in the testes is disrupted. According to Arafa et al. [39], HES prevents pyknosis, necrobiotic changes, and chromatolysis in spermatocyte nuclei in the seminiferous tubules of rats exposed to benzo[α]pyrene. Severe necrotic and degenerative alterations were observed in the reproductive systems of methotrexate-treated male mice, whereas HES was found to lower the degree of necrotic damage [38].

## 5. Conclusions

The results of this study revealed that ROS attacks on the testicular system can damage germ cell membranes, causing sperm malfunction, histopathological changes, and cell death. HES administered at a dose of 100 mg/kg/day effectively reduced GLP-induced testicular damage in the rats, maintaining testicular structures. We need modern technological research to evaluate the clinical usefulness of GLP and HES.

## Figures and Tables

**Table 1:** Spermatozoon motility, abnormality, and testosterone levels, Mean ± SEM.

Groups	Motility %	Testosterone ng/L	Abnormal Sperm Counts			
			Head %	Middle %	Tail %	Total %
C	76.42 ± 2.03 ^a^	433.99 ± 12.03	4.07 ± 0.66 ^b^	5.57 ± 0.74 ^a^	8.53 ± 1.83 ^b^	18.64 ± 2.26 ^b^
HES	81.00 ± 2.05 ^a^	437.76 ± 18.70	2.64 ± 0.50 ^b^	2.21 ± 0.39 ^b^	6.57 ± 0.39 ^b^	11.50 ± 0.99 ^c^
GLP	47.57 ± 8.93 ^b^	470.93 ± 24.79	6.35 ± 0.72 ^a^	6.92 ± 0.81 ^a^	21.50 ± 1.56 ^a^	34.92 ± 1.17 ^a^
GLP + HES	75.71 ± 3.56 ^a^	440.55 ± 11.16	7.71 ± 0.82 ^a^	3.71 ± 0.47 ^b^	9.50 ± 0.99 ^b^	21.07 ± 1.61 ^b^
*p*	*	NS	*	*	*	*

On the same column, the means marked with different superscripts (a, b, c) differ significantly (* *p* < 0.05). NS: not significant, C: control, GLP: glyphosate, HES: Hesperidin.

**Table 2:** Sperm membrane integrity values, Mean ± SEM.

Groups	H+/E− %	H−/E− %	H+/E+ %	H−/E+ %
C	16.71 ± 2.11	26.57 ± 3.37 ^b^	22.71 ± 2.64 ^a^	34.00 ± 3.39
HES	17.42 ± 1.92	38.00 ± 2.87 ^a^	12.42 ± 1.17 ^b^	31.42 ± 3.28
GLP	16.42 ± 1.39	18.42 ± 1.65 ^c^	28.71 ± 3.89 ^a^	36.42 ± 3.83
GLP + HES	18.00 ± 1.86	31.85 ± 2.85 ^ab^	13.57 ± 1.52 ^b^	35.14 ± 3.08
*p*	NS	*	*	NS

On the same column, the means marked with different superscripts (a, b, c) differ significantly (* *p* < 0.05). NS: not significant, H−/E−, tail not swollen and head white; H+/E−, tail swollen and head white; H−/E+, tail not swollen and head red; H+/E+, tail swollen and head red. C: control, GLP: glyphosate, HES: Hesperidin.

**Table 3:** Testicular and epididymis weight values, Mean ± SEM.

Groups	Left Testis	Right Testis	Left Epididymis	Right Epididymis
C	6.25 ± 0.11 ^b^	3.55 ± 0.30	6.28 ± 0.12	4.07 ± 0.24 ^ab^
HES	6.57 ± 0.19 ^ab^	3.72 ± 0.33	6.61 ± 0.20	3.61 ± 0.13 ^b^
GLP	7.07 ± 0.17 ^a^	3.88 ± 0.16	6.40 ± 0.49	4.20 ± 0.23 ^a^
GLP + HES	6.86 ± 0.20 ^a^	4.38 ± 0.41	6.90 ± 0.18	4.24 ± 0.09 ^a^
*p*	*	NS	NS	*

On the same column, the means marked with different superscripts (a, b, c) differ significantly (* *p* < 0.05). NS: not significant, C: control, GLP: glyphosate, HES: Hesperidin.

**Table 4:** Oxidative stress parameters, Mean ± SEM.

Groups	MDA (nmol/mL)	GSH (nmol/mL)	TAS (mmol/L)	TOS (μmol/L)	OSI (TOS/TAS × 100)
C	7.12 ± 0.30 ^b^	7.12 ± 0.30 ^a^	0.99 ± 0.03 ^a^	12.44 ± 0.50 ^b^	127.36 ± 8.57 ^b^
HES	8.71 ± 0.60 ^a^	4.35 ± 0.30 ^b^	0.81 ± 0.02 ^b^	11.38 ± 0.49 ^b^	141.60 ± 8.30 ^b^
GLP	9.23 ± 0.49 ^a^	2.31 ± 0.12 ^c^	0.77 ± 0.01 ^b^	17.49 ± 1.44 ^a^	225.66 ± 17.60 ^a^
GLP + HES	6.94 ± 0.29 ^b^	2.31 ± 0.09 ^c^	0.81 ± 0.03 ^b^	10.83 ± 1.84 ^b^	134.82 ± 25.83 ^b^
*p*	*	*	*	*	*

On the same column, the means marked with different superscripts (a, b, c) differ significantly (* *p* < 0.05). C: control, GLP: glyphosate, HES: Hesperidin. MDA: Malondialdehyde, GSH: glutathione, TAS: total antioxidant status, TOS: total oxidant status, OSI: oxidative stress index.

**Table 5:** DNA damage values, Mean ± SEM.

Groups	Tail Length (μm/s)	Tail DNA (%)	Tail Moment (μm/s)
C	31.32 ± 0.28 ^c^	66.73 ± 0.93	21.51 ± 0.22 ^c^
HES	34.02 ± 0.34 ^b^	67.71 ± 0.52	23.57 ± 0.23 ^b^
GLP	38.13 ± 0.69 ^a^	67.46 ± 0.44	26.32 ± 0.67 ^a^
GLP + HES	33.69 ± 0.77 ^b^	68.64 ± 0.62	23.28 ± 0.49 ^b^
*p*	*	NS	*

On the same column, the means marked with different superscripts (a, b, c) differ significantly (* *p* <0.05). NS: not significant, C: control, GLP: glyphosate, HES: Hesperidin.

**Table 6:** Results of histopathological analyses on effects of HES against GLP-induced testicular toxicity in male rats ($\bar{x}$ ± S$\bar{x}$).

Groups	Immature Germinal Cells in Tubular Lumen	Epithelial Degeneration	Vacuolization in Seminiferous Tubules	Atrophy in Seminiferous Tubules	Congestion in Testes
C	0.01± 0.01 ^b^	0.29 ± 0.06 ^b^	0.49 ± 0.07 ^b^	0.06 ± 0.03 ^b^	0.14 ± 0.04 ^b^
HES	0.06 ± 0.03 ^b^	0.43 ± 0.07 ^b^	0.54 ± 0.08 ^b^	0.01 ± 0.01 ^b^	0.19 ± 0.05 ^b^
GLP	0.46 ± 0.08 ^a^	1.16 ± 0.10 ^a^	1.50 ± 0.09 ^a^	0.27 ± 0.05 ^a^	0.67 ± 0.09 ^a^
GLP + HES	0.06 ± 0.20 ^b^	0.31 ± 0.06 ^b^	0.54 ± 0.08 ^b^	0.01 ± 0.01 ^b^	0.24 ± 0.05 ^b^
*p*	***	***	***	***	***

On the same column, the means marked with different superscripts (a, b, c) differ significantly. *** *p* < 0.001, C: control, HES: Hesperidin, GLP: glyphosate, n: number of rats, $\bar{x}$: mean, S$\bar{x}$: standard error.

**Table 7:** Effectsof HES on histomorphometric parameters of seminiferous tubules (height and diameter; µm) against GLP-induced toxicity in male rats ($\bar{x}$ ± S$\bar{x}$).

Groups	Stage VII-VIII STDs (µm)	Stage VII-VIII SEHs (µm)	Stage XIV Tubules (%)
C	291.91 ± 2.52	62.65 ± 0.52 ^a^	5.57 ± 0.45
HES	297.42 ± 2.94	62.89 ± 0.50 ^a^	6.64 ± 0.55
GLP	290.17 ± 2.88	59.90 ± 0.56 ^b^	5.07 ± 0.63
GLP + HES	294.69 ± 2.86	63.66 ± 0.58 ^a^	5.64 ± 0.68
*p*	NS	***	NS

On the same column, the means marked with different superscripts (a, b) differ significantly. ***: *p* < 0.001, NS: not significant, C: control, HES: Hesperidin, GLP: Glyphosate, STD: seminiferous tubule diameters, SEH: seminiferous epithelial heights, $\bar{x}$: mean, S$\bar{x}$: standard error.

## Data Availability

The data supporting this study’s findings are available from the corresponding author upon reasonable request.
